# Exploring the Role of Locus Coeruleus in Alzheimer’s Disease: a Comprehensive Update on MRI Studies and Implications

**DOI:** 10.1007/s11910-023-01324-9

**Published:** 2023-12-08

**Authors:** Alessandro Galgani, Filippo Sean Giorgi

**Affiliations:** Department of Translational Research and of New Surgical and Medical Technologies School of Medicine, University of Pisa, Via Roma 55, 56126 Pisa, Italy

**Keywords:** Locus coeruleus, Alzheimer’s disease, Magnetic resonance imaging, Ageing, Noradrenaline, Neurodegeneration

## Abstract

**Purpose of Review:**

Performing a thorough review of magnetic resonance imaging (MRI) studies assessing locus coeruleus (LC) integrity in ageing and Alzheimer’s disease (AD), and contextualizing them with current preclinical and neuropathological literature.

**Recent Findings:**

MRI successfully detected LC alterations in ageing and AD, identifying degenerative phenomena involving this nucleus even in the prodromal stages of the disorder. The degree of LC disruption was also associated with the severity of AD cortical pathology, cognitive and behavioral impairment, and the risk of clinical progression.

**Summary:**

Locus coeruleus-MRI has proved to be a useful tool to assess the integrity of the central noradrenergic system in vivo in humans. It allowed to test in patients preclinical and experimental hypothesis, thus confirming the specific and marked involvement of the LC in AD and its key pathogenetic role. Locus coeruleus-MRI–related data might represent the theoretical basis on which to start developing noradrenergic drugs to target AD.

## Introduction

In recent years, the locus coeruleus (LC) has been receiving increasing attention in Alzheimer’s disease (AD) research [1•]. The LC is the main hub of the brain noradrenergic (NA) system and provides the NA innervation for the whole cortical mantle and for many subcortical structures [2]. Its wide and diffuse projections form the morphological substrate for the many functions the LC plays in the central nervous system (CNS) [3]. From a neurophysiological point of view, the LC-NA system takes part in the sleep/wake circuitry [4], as well as in modulating cortical networks implied in cognitive functions, mainly attention and memory [5, 6]. At the cellular level, NA is crucial in maintaining brain homeostasis, regulating the neurovascular coupling, promoting blood–brain barrier (BBB) integrity, and modulating glial cell activity [7, 8].

### A Noradrenergic Hypothesis for Alzheimer’s Disease

The LC is a tiny cluster of NA cells, with a neuronal population ranging from 20,000 to 60,000, and it is placed just below the floor of the fourth ventricle of the rostral pons [9]. Because of its location, the LC is particularly exposed to neurotoxins coming from the blood and cerebrospinal fluid (CSF) streams [10]. Furthermore, the significant production of oxidative species during NA metabolism, combined with the LC susceptibility to damage due to its exclusive blood supply through pontine arterioles that are highly prone to arteriolosclerosis, might facilitate the deterioration LC with age [10, 11]. Indeed, for a long time, it was believed that a physiological age–dependent degeneration might be responsible for the spontaneous disruption of this nucleus [12]. However, recent neuropathological data has strongly associated this phenomenon with the occurrence of neurodegenerative disease, particularly AD pathology rather than with normal ageing. According to the revised Braak’s staging for neurofibrillary pathology in AD, the LC is the first brain structure to show specific alterations, even decades before the clinical onset of the disease [13]. In parallel, it has been clearly shown that the neuronal death occurring in LC is invariably related to the AD pathology burden in the forebrain, with small or no damage observed in pathology-free brains, regardless of the age of the analyzed subject [14].

Such a recontextualization of LC degeneration, shifting from being viewed as an accidental phenomenon to a pathology-specific feature, coincided with experimental evidence showing how the loss of LC-NA innervation might promote and worsen AD pathogenesis [15]. The experimental lesion of the LC in AD animal model has been associated with higher amyloid burden [16], increased neuroinflammation [17, 18], and neurovascular alterations [19]. Therefore, preclinical data point toward a crucial role of LC degeneration both in the onset and progression of AD pathogenesis [20, 21].

### Locus Coeruleus Magnetic Resonance Imaging

The clinical challenge of this “NA hypothesis” has been addressed thanks to the development of a new neuroimaging tool, the LC-sensitive Magnetic Resonance Imaging (LC-MRI) [22•]. Despite its small size, the LC can be visualized by MRI using high-frequency pulsed sequences [23, 24], which make it appear hyperintense in brain scans of the upper pons. The source of the LC signal is still a matter of debate. On the one hand, the occurrence of a pigmented molecule, i.e., neuromelanin (NM), which can bind metallic ions, such as iron and copper, suggested that NA cells in the LC possess paramagnetic properties making them appear hyperintense in T1-weighted sequences [25, 26]. On the other hand, several authors proposed a possible role of the magnetization transfer (MT) effect [27, 28], due to the peculiar anatomy of the LC itself, which consists of a small cluster of densely packed cells intermingled with white matter bundles [29, 30]. To address this issue, an MRI study was performed in mice lacking the gene coding for dopamine-β-hydroxylase (DBH) [31], the key enzyme for NA and NM synthesis. When analyzed with MT-MRI, these animals still showed a LC-related signal [31]. Even though that study strongly suggested that NM is not the source of the signal, certain features observed in LC-MRI align more closely with the NM hypothesis; in particular, the age-dependent increase in LC intensity observed during the first half of the lifespan [32–34] is more in line with the well-known progressive accumulation of NM in NA cells [25] than with morphological changes in the LC itself, which reaches its complete differentiation in the early post-natal years [35]. Whatever it might be the main source of LC-MRI hyperintensity, this tool has been extensively and profitably used in a variety of fields of neuroscience research, ranging from neurodegenerative disorders to psychiatric conditions [20, 36, 37].

Many studies have been performed in AD, exploring the association between MRI-assessed LC integrity and AD pathological and clinical features. A certain degree of methodological variability might make it sometimes challenging to directly compare these studies, in terms of MRI scans used (3 Tesla vs. 7 Tesla), MRI protocols applied (T1-weighted or MT sequences), and post-processing analyses (native space– vs. template space–based approaches) [38]. Nonetheless, this does not affect the main goal of this review, which is to summarize the remarkable amount of data collected using LC-MRI in AD patients thus far and integrate it within the context of the existing preclinical and neuropathological literature.

## LC Alteration in Ageing Is Not Harmless: Insight from LC-MRI Studies in Cognitively Unimpaired Elderlies

As mentioned earlier, initial neuropathological studies in elderly and AD patients did not identify a pathological specificity of LC degeneration but rather led to the hypothesis of a spontaneous age-dependent disruption of the nucleus [12]. However, more recent and robust studies have clearly shown that such a phenomenon, in the absence of other concomitant neurological disorders, is invariably associated with the onset and progression of AD pathology in the forebrain [13, 14]. Clinical data from LC-MRI studies are in line with the latter interpretation (Table 1), once taken together and coherently analyzed.

**Table 1:** Locus coeruleus magnetic resonance imaging (LC-MRI) studies in cognitively unimpaired elderlies

Year	Author	MRI scan	Population	Age	Main findings
2019	Dahl et al. [39]	3 T	Y 66 E 228	72.3 (4.1)	In elderlies, rostral LC signal is directly associated with better memory performances
2019	Liu et al. [40]	3 T	605	18–88	LC signal changes with age following an inverse U pattern. In elderlies, rostral LC signal is directly associated with better memory performances
2020	Liu et al. [41]	3 T	603	18–88	LC signal is associated with global cognitive and behavioral performances. Models in which all cognitive and behavioral items are pooled together fit better with LC variability than the ones in which NA-dependent and independent ones are separated
2021	Giorgi et al. [42]	3 T	53	71.7 (4.7)	No association between LC signal and age is found
2021	Bachman et al. [43]	3 T	Y 67 E 229	72.3 (4.0)	In elderlies, LC signal is directly associated with cortical thickness of frontoparietal cortex, belonging to cognitive neuronal network modulated by the LC-NA system
2021	Van Egroo et al. [44]	7 T	72	65.2 (8.2)	LC signal is not associated with global sleep quality, but with the amount of nocturnal awakening. This relationship is stronger in subjects with higher level of plasmatic pTau (always within the normal range)
2022	Elman et al. [45]	3 T	435	62–71	LC signal is directly associated with structural complexity of the cortex (assessed through cortical DTI) but not with cortical thickness
2023	Bell et al. [46]	3 T	381 (SCI)	67.6 (2.6)	In SCI subjects, the rostral LC signal is directly associated with cognitive performances (memory, executive and visuospatial)
2023	Al Haddad et al. [47]	3 T	152	53–86	No association between LC signal and age is found
2023	Van Egroo et al. [48••]	7 T	99	59.9 (13.1)	The rostral LC signal is negatively associated with plasmatic levels of pTau, while only limited or no correlations are observed for other plasmatic biomarkers, amyloid ones included. The rostral part is the most vulnerable region of the LC to tau pathology in AD
2023	Dahl et al. [49••]	3 T	Y 69 E 251	72.4 (4.0)	LC signal is associated with better episodic memory and predicts variation in episodic memory over time

Age is reported either as mean and standard deviation (between round brackets) or range, according to data provided in each article; in the case of young and elderlies groups, only the age of elderly ones is reported

*LC* locus coeruleus, *E* elderly subjects, *NA* noradrenergic, *Y* young subjects, *pTau* hyperphosphorylated tau, *SCI* subjectively cognitive impaired, *T* tesla

Indeed, two studies, in which cognitively unimpaired elderlies were recruited after a detailed neurological and cognitive assessment and submitted to LC-MRI, failed to detect LC alterations with ageing in healthy subjects [42, 47], as no inverse associations of the LC-MRI signal with age [42, 47] or cognitive performances were found [42]. On the contrary, in more studies, an age-dependent reduction of LC signal in healthy elderlies was proposed [39–41, 43, 45, 46, 49••]. In particular, analysis of larger cohorts of subjects, in which the authors explored LC features across the entire lifespan, found that the LC signal progressively increases up to the 5th–6th decades of life and then starts to decrease in subsequent years [39–41]. Even though these pieces of evidence appear to be in contrast with our premises, it is worth noting that such variability was not devoid of neurobiological correlates. Firstly, the reduction of LC signal mainly involved its rostral part [39, 40], which is a subregion of this nucleus key for AD pathophysiology, as we will discuss later (see the paragraph “LC degeneration in AD: an early event with prognostic value). Secondly, in the above-quoted cohorts, the reduction of the LC signal was consistently associated with a decline in cognitive performances or disruption in brain morphological integrity. Indeed, LC integrity has also been directly associated with the cortical thickness of parietal, frontal, and occipital areas [43] and with the complexity of cortical microstructure, assessed using diffusion tensor imaging (DTI) as a proxy [45]. These data are paralleled by the results of tractography studies, which showed a reduction of structural integrity of LC-NA projections to the forebrain in these types of subjects [50, 51]. Altogether, the above-reported LC-MRI data suggests that a loss of integrity of LC in elderlies is associated with morphological signs of precocious brain ageing. Plini and colleagues further support this interpretation [52]. They explored the relation between LC-MRI signal and a composite score of brain deterioration relative to chronological age and found that a lower LC signal was associated with more evident signs of pathological brain ageing [52]. In line with structural findings, also cognitive performances were directly associated to LC integrity. A lower LC signal was related to worse memory performances [39, 40, 49••], global cognition and behavioral status [41] and was also able to predict a further deterioration of memory overtime [49••]. These structural findings are supported by functional MRI (fMRI) studies, which disclosed a reduction of LC activation in elderlies with worse cognitive performances [53, 54].

Thus, it appears that the age-dependent reduction reported by many authors should not be interpreted as a mere physiological variation occurring in elderlies, but rather as an early hint of an underlying pathological process. To explore this phenomenon, Liu and colleagues, in a study published in 2020 [41], investigated the association between the LC-MRI signal and cognitive and behavioral performances. They built two different models, in which the analysis was performed either considering all the cognitive and behavioral scores together or splitting them according to the modulation LC-NA might exert on them. Unexpectedly, they found that the best-fitting model was the former, rather than the latter [41]. Thus, it might be suggested that the LC loss of integrity might not directly influence the deterioration of the cognitive status, since not only NA-dependent functions are involved, but that such a relation might be mediated by something else, i.e., AD pathology. This interpretation is supported by the study by Van Egroo and colleagues, which in 2023 published a report on cognitively unimpaired elderlies in which they assessed LC integrity through 7 T MRI scan and explored its association with plasmatic hyperphosphorylated tau (pTau) levels, considered a proxy for cortical AD–related tau pathology [48••]. They found that the progressive reduction of LC signal observed in elderlies is associated with a concomitant increase of plasmatic pTau levels, further confirming the close relationship between LC disruption and AD pathology [48••]. Remarkably, and in line with this data, another study found that in elderly subjects lacking AD cortical pathology, as detailed through Positron Emission Tomography (PET) with both amyloid and tau tracers, the negative association between LC integrity and age disappeared [55••].

## LC Degeneration in AD: an Early Event with Prognostic Value

With the only exception of the first study published on the topic [56], all the published studies in the field converge in showing that LC-MRI—when properly performed—allows to successfully detect LC degeneration in AD [44, 52, 55••, 57–62, 63••, 64••, 65–68, 69••, 70, 71]. Perfectly in line with neuropathological data [72], these neuroimaging studies found a significant reduction of LC signal occurring not only in the most severe cases of AD (dementia) but also in the prodromal phases of the disease, clinically defined as Mild Cognitive Impairment (MCI) [44, 52, 55••, 57–62, 63••, 64••, 65–68, 69••, 70, 71]. This confirmed in vivo that LC involvement is an early feature of AD progression and suggested that the loss of NA neuroprotective effect might begin to influence AD pathogenesis before the clinical onset of the disease.

Interestingly, many of those studies highlighted that the most severely damaged region of LC was its rostral part [59, 66, 69••, 70, 71]. It is well known that in terms of topographical distribution within the LC itself, the NA neurons whose projections target the limbic cortex are those placed in the rostral part of the nucleus [15, 73, 74]. Limbic structures, such as the entorhinal cortex and the hippocampus, are those brain areas that are most severely involved by AD cortical pathology [75] and whose impairment mainly drives its pathophysiology [76, 77]. Thus, the selective involvement of this LC subregion, which has not been observed in other forms of degenerative cognitive decline [70], further supports the attribution of a pathogenic role to LC degeneration in AD. As already said, experimental studies showed that the lesion of LC in mouse models causes the exacerbation of AD pathology, by increasing the amyloid burden [16], promoting neurovascular alterations [7, 19] and exacerbating aberrant neuroinflammatory processes [17, 18].

The clinical implications of these pieces of data should be that AD patients with lower LC signal at MRI should present a poorer prognosis, with a faster cognitive decline and more severe pathological alterations. Some neuroimaging studies supported this hypothesis [52, 62, 67, 68, 69••, 78]. In particular, some authors found a direct association between LC degeneration and cortical thickness [68], structural complexity [68, 78], and the degree of brain atrophy [52] in AD patients, and in one study, LC signal was directly associated with cortical hypometabolism in the frontoparietal cortices, assessed through fluorodeoxyglucose (FDG)-PET [67]. Indeed, MRI-assessed cortical atrophy/derangement and FDG uptake reduction at brain PET are considered neuroimaging prognostic biomarkers, as they reflect neuronal death occurring in AD brains [79].

The clinical confirmation of the prognostic role of LC-MRI alteration in MCI patients was provided by Galgani and colleagues in 2023. In this paper, they reported the results of a quite prolonged longitudinal LC-MRI study [69••]. They found that MCI subjects who progressed to dementia during a 30-month follow-up showed at baseline a lower signal in the rostral LC when compared to the ones who remained cognitively stable [69••].

### The Association Between LC Disruption and AD Pathology: the Link with Tau

Several authors aimed to dissect the association between LC degeneration and AD pathological hallmarks, i.e., tau and amyloid pathology [55••, 59, 63••, 64••, 66]. The first papers addressing this aspect found none or only a limited association between the LC signal and amyloid pathology, assessed either through CSF analysis [59] or amyloid PET [60]. However, in 2021, Jacobs and colleagues published a very interesting study, in which they explored the integrity of LC by MRI in a cohort of both cognitively intact and impaired elderlies, whose AD pathology had been assessed through amyloid and tau PET scans [55••]. They found that the LC signal was indirectly linked to the brain uptake of both tracers, with a significant distinction between the two associations; while the LC-tau relation showed a topographical distribution, with greater strength for entorhinal and temporal cortices compared to the frontoparietal regions, the LC-amyloid association was diffuse and spread across the entire cortical mantle [55••]. In line with these results, Cassidy and colleagues performed a similar study in 2022, in which they found that the reduction of LC-MRI signal was indirectly associated with patients’ Braak stage, extrapolated from tau PET scans [63••]. The findings of these two studies suggest that the LC disruption occurring in AD might be directly associated with neurofibrillary tangles (NFT) pathology, while the link with amyloid accumulation might be more indirect and mediated by other pathological mechanisms. Investigations performed in autosomal dominant AD (ADAD) cases pointed toward the same hypotheses. In 2021, Dahl and colleagues confirmed the indirect association between LC integrity and abnormal tau cortical deposition in a group of ADAD cases, both still cognitively intact and already cognitively impaired [66]. When analyzing another group of ADAD subjects, Jacobs and colleagues obtained similar data, but they demonstrated also that the LC-tau association occurred independently from amyloid pathology and could predict further tau cortical accumulation [64••]. Further in vivo support for the association between LC degeneration and tau pathology in AD came from two fMRI studies, in which the authors found that LC loss of activation was directly related to tau accumulation in the entorhinal cortex and cognitive decline [80], while amyloid accumulation had a more pronounced impact as a group differentiator (i.e., distinguishing between amyloid-positive and negative groups) rather than directly correlating with LC dysfunction [81].

Altogether, these studies highlighted how the progressive LC degeneration occurring in AD is related to forebrain tau pathology, a trend that was already shown also in cognitively intact elderlies [48••]. These findings align seamlessly with previous neuropathological studies demonstrating that LC itself is precociously involved in AD tau pathology and might promote the subsequent cortical accumulation of NFT. In the 2011 revised version of the Braak NFT staging system, not only is the LC identified as the initial brain structure to display AD-related pathological alterations, but these are specifically characterized by pTau accumulation within the NA cells [13]. The accumulation of pTau in the LC follows a time-dependent trend, increasing with ageing [48••]. Interestingly, in line with what was mentioned in the previous paragraph, the rostral part of the LC is the subregion most vulnerable to pTau deposit [48••]. PTau accumulation then turns into proper NFT pathology and leads to neuronal death and LC degeneration with the progression of Braak stages and AD [14, 48••, 55••]. This strong association between LC degeneration and tau accumulation supports the hypothesis that this nucleus might not only be particularly vulnerable to tau pathology, but even its site of origin, promoting both pTau formation and spreading to the whole CNS exploiting its NA projections [82]. Experimental data support this assumption. Indeed, in vitro findings highlighted a metabolic association between NA cells and tau protein turnover, suggesting that NA metabolites might promote a specific cleavage of tau which might be then more susceptible to hyperphosphorylation and accumulation [83, 84]. Moreover, pTau possesses well-known seeding properties [85]; in a transgenic tau pathology mouse model, the injection of synthetic pTau in the LC led to its diffusion in the whole CNS through the NA fibers originating from the nucleus [86], and the experimental lesion of the LC caused a marked worsening of the same tau pathology [87]. Hence, the existing data strongly substantiate the pathological and pathogenic link between LC involvement in AD and tau pathology [88] and, thus, suggest that LC-MRI might be considered an actual proxy of tau accumulation in vivo [48••].

Regarding the relation of LC impairment with amyloid pathology, this might occur with the mediation of tau-related mechanisms. The disruption of LC-NA projections due to neuronal death and tau accumulation might cause the loss of a neuroprotective effect of NA onto microglial cells and the neurovascular unit [7, 17, 19, 89]. Theoretically, this might lead to amyloid accumulation by compromising its clearance pathways on multiple levels, i.e., both by reducing its phagocytosis and degradation by microglia [16, 18], and by hampering its disposal through the BBB [7, 90].

### LC-MRI as a Tool to Explore the Effects of the LC-NA System Impairment on AD Clinical Features

Using MRI to explore the integrity of LC in elderlies, either cognitively intact or suffering from AD, allowed not only to confirm in vivo its crucial involvement in this neurodegenerative disorder, but also to highlight its association with cognitive and behavioral symptoms. As said, the LC-NA system plays multiple roles in the regulation of cognition and behavior [2].

The clinical core of typical AD is the onset of progressive decline in episodic memory, which is subsequently accompanied by changes in other cognitive domains, including executive functions, visuospatial ability and speech [91]. Even though many MRI studies point toward a mediation of AD pathology in the association between LC disruption and cognitive impairment [41, 55••, 64••], one cannot exclude that the loss of NA innervation might exert a direct effect as well.

The cognitive function most influenced by LC-NA modulation is likely attention, intended as the ability to focus on a specific and salient stimulus, switching the focus from a previous to a novel one, and, at the same time, correctly orienting the subjects into the outer (or inner) environment [92, 93]. This cognitive ability is one of the earliest to be affected in AD [94]. Some studies investigated the degree of such impairment in the light of LC degeneration and confirmed the occurrence of this association both by assessing attention ability through neuropsychological testing [51, 52, 69••, 71] and by exploring the integrity of the correspondent frontoparietal cortical network [67, 78].

However, in all the LC-MRI studies in which there was a concomitant cognitive assessment, a consistent correlation emerged between LC integrity and memory performance, spanning both verbal and visuospatial memory [55••, 60, 61, 64••, 66, 69••, 71]. Even though this specific relation might be mediated by ongoing AD pathology, and in particular the tau-related one [64••], preclinical studies suggest that also the NA loss might play a specific role by itself [95]. On a macroscale level, experimental lesion of LC impacts memory performance in animal models [18, 87, 96, 97], whereas, on a microscale level, the loss of NA innervation has been linked to reduced hippocampal neurogenesis and impaired synaptic plasticity [96, 98, 99]. Thus, it might be that LC degeneration could affect the hippocampus with two different mechanisms in AD, i.e., both exacerbating amyloid pathology and impairing neuronal homeostasis and function.

AD is also characterized by behavioral changes, ranging from prodromal mood alterations to frank psychiatric disturbances occurring later in the course of the disease [100]. The LC-NA system takes part in the modulation of mood and behavior [101–103], and some investigations have utilized LC-MRI in late-life depression patients, although the results have been limited [104, 105]. In their study, Cassidy and colleagues found an association between neuropsychiatric alterations and LC integrity in AD patients [63••]; interestingly, their data showed a direct association of loss of impulse control with LC signal, meaning that the higher the signal in AD patients, the more severe the behavioral symptoms tended to be [63••].

Finally, a subset of LC-MRI studies investigated the influence of LC disruption on circadian rhythm alteration and disturbances in sleep patterns [71, 106, 107], which occur very often in AD patients and whose onset may even precede the appearance of cognitive symptoms [108]. Even though those studies were performed in cognitively intact elderlies or MCI subjects, they shed some light on this additional pathophysiological mechanism of AD. In 2021, Elman and colleagues observed an association between LC signal and daytime dysfunction, the latter assessed by an ad hoc questionnaire [71]. In the same year, Van Egroo et al. published a report in which they evaluated the sleep quality of cognitively intact elderlies, whose neuropathological background had been assessed through the plasmatic level of pTau [44]. They found no association between sleep quality and LC signal but rather an inverse correlation with the number of nocturnal awakenings, which was stronger in subjects with higher levels of plasmatic pTau, i.e., more likely to be affected by AD [44]. This piece of data becomes even more interesting considering the results of another study, published by Koshmanova and colleagues in 2023 [107]. In a group of elderly subjects, they found that LC signal was directly associated with sleep quality (in contrast with [44]) but, at the same time, that a higher LC activity during wake, assessed through fMRI, was associated with worse sleep quality and lower power of EEG theta band during REM sleep [107]. Altogether, these studies suggest an in vivo detectable association between LC-MRI integrity and sleep, but with a double pattern. On the one side, the progressive loss of integrity of LC alters daytime circadian rhythms [71] and impairs sleep quality [107], all processes in which the LC-NA system plays a key role [109]. On the other side, the disruption of this nucleus might be also associated with an abnormal and excessive LC-NA activation, which in turn might be responsible for “irritative symptoms” [44], as suggested by Koshmanova and colleagues [107], and also by Cassidy et al. for behavioral dyscontrol [63••].

## Conclusive Remarks

In this paper, we reviewed the extensive body of LC-MRI studies conducted on AD patients and elderly subjects (Table 2), framing their findings within the context of current experimental and neuropathological literature. We found that neuroimaging studies match the *post-mortem* ones, clearly detecting LC degeneration in the earliest stages of clinical AD [44, 52, 55••, 57–62, 63••, 64••, 65–68, 69••, 70, 71]. Furthermore, human studies are in line with experimental data, supporting a possible role of LC disruption in AD pathogenesis and clinical progression [52, 62, 67, 68, 69••, 78]. Altogether, LC-MRI studies have supported the “NA hypothesis” of AD in vivo*.* This opens new insights, with implications for both diagnosis and treatment [1•].

**Table 2:** Locus coeruleus magnetic resonance imaging (LC-MRI) studies in Alzheimer’s disease patients

Year	Author	MRI scan	Population	Main findings
2019	Betts et al. [59]	3 T	HC 25 SCI 21 MCI 16 ADD 11	LC signal is significantly lower in ADD when compared to HC, while no differences are found with MCI or SCI subjects. LC signal inversely correlates with CSF amyloid parameters
2019	Olivieri et al. [60]	3 T	HC 17 AD 37 (21 typical, 17 atypical)	LC signal is significantly lower in AD patients than in HC, both considering typical and atypical AD, either in the prodromal or mild dementia phase. LC signal directly correlates with episodic memory performances
2021	Hou et al. [61]	3 T	HC 24 AD 24	LC signal is significantly lower in AD when compared to HC and correlates with global cognitive scores
2021	Elman et al. [71]	3 T	HC 373 aMCI 36 naMCI 22	A lower LC signal increases the risk of the aMCI diagnosis. Moreover, LC signal is inversely associated with the amount of circadian alterations and daytime disfunction. Such a relation is stronger in aMCI subjects
2021	Jacobs et al. [55••]	3 T	HC 138 AD 36 (+ 15 Y and 32 M)	LC signal is significantly lower in AD when compared to HC and it is inversely associated with both tau and amyloid cortical burden. The association LC-tau pathology shows a topographical distribution, being stronger in the entorhinal cortex and temporal cortex than in parietal and frontal cortices. LC signal correlates with memory performances, mediated by cortical tau pathology. No association with age is found in subjects negative for both tau and amyloid pathology
2021	Plini et al. [52]	3 T	HC 395 MCI 156 ADD 135	LC signal is significantly lower both in ADD and MCI patients when compared to HC, and it is associated with more severe chrono-dependent brain atrophy
2021	Dahl et al. [66]	3 T	ADAD − 9 ADAD + CU 5 ADAD + CI 4	LC signal is significantly lower in ADAD + subjects than in ADAD − ones, with the middle-rostral part of the nucleus being more severely involved. In ADAD + , LC signal is associated with cortical tau pathology and with cognitive performances
2022	Cassidy et al. [63••]	3 T	HC 118 MCI 44 AD 28	LC signal is significantly lower in ADD when compared to HC. No difference with MCI subjects is found. Tau-positive subjects shows significantly lower LC signal than tau-negative ones. LC signal is negatively correlated to the PET-assessed Braak stage
2022	Li et al. [65]	3 T	HC 25 MCI 15 ADD 27	LC signal is significantly lower in ADD patients but not in MCI subjects, when compared to HC
2023	Jacobs et al. [64••]	3 T	27 PSEN + 27 PSEN −	LC signal is significantly lower in cognitively impaired PSEN + patients than in cognitively intact PSEN + individuals or PSEN − subjects. LC signal is inversely associated to age in PSEN + individuals, regardless of their cognitive status and their cortical amyloid and tau burden. LC signal is inversely associated with tau entorhinal cortex deposition in PSEN + , both cognitively intact and impaired. The same association is found for global cortical amyloid burden. Tau pathology mediates the association between LC signal and memory performances. LC signal predicts further tau cortical accumulation in PSEN + individuals
2023	Aghakanyan et al. [67]	3 T	HC 15 30 MCI 13 ADD	LC signal is significantly lower in cognitively impaired patients than in cognitively intact individuals. LC signal is directly associated with cortical glucidic metabolism (assessed through FDG-PET) in frontoparietal regions of MCI and ADD patients
2023	Liu et al. [68]	3 T	HC 25 AD 27	LC signal is directly associated with cortical thickness and structural complexity (assessed using fractal dimension as a proxy)
2023	Tang et al. [78]	3 T	HC 161 SCI 99 MCI 131	LC signal is significantly lower in MCI individuals, when compared to SCI subjects or HC. LC-related structural covariance is altered in MCI individuals, involving the brain region of the default-mode network and salience networks
2023	Galgani et al. [69••]	3 T	HC 53 MCI 77 ADD 34	LC signal is significantly lower in ADD and cMCI patients when compared to ncMCI and HC. MCI subjects with lower LC signal show a higher risk to convert to dementia (30 months longitudinal follow-up). LC signal is directly associated with global cognitive performances
2023	Galgani et al. [70]	3 T	HC 53, ADD 34, DLB 11	LC signal is significantly lower in ADD and DLB patients, when compared to HC, with different topographical patterns. In ADD, the rostral region of the LC is more severely involved, while in DLB, a global degeneration can be found

*AD* Alzheimer’s disease, *ADAD* autosomal dominant Alzheimer’s disease, *ADD* Alzheimer’s disease dementia, *aMCI* amnestic mild cognitive impairment, *cMCI* mild cognitively impaired subjects converted to dementia, *CI* cognitively impaired, *CU* cognitively unimpaired, *DLB* dementia with Lewy body, *FDG* fluorodeoxyglucose, *HC* healthy controls, *LC* locus coeruleus, *M* middle-aged subjects, *MCI* mild cognitively impaired, *NA* noradrenergic, naMCI non-amnestic mild cognitive impairment, *ncMCI* mild cognitively impaired subjects not converted to dementia, *PET* positron emission tomography, *pTau* hyperphosphorylated tau, *PSEN* presenelin 1, *SCI* subjectively cognitive impaired, *T* tesla, *Y* young subjects

LC-MRI as a neuroimaging tool has been attracting growing interest in the clinical field [22•], even though the notable variability that characterizes the LC signal might hamper its use in the routinary diagnostic work-up as an individual biomarker. However, it remains a promising and valuable technique when evaluated together with other potential biomarkers in a single subject, and as a group biomarker for clinical research [22•].

The primary objective of this emerging narrative is to introduce a new pharmacological approach in AD, aiming to target the LC-NA system, not only to halt the ongoing neurodegenerative process but also to restore the brain’s physiological homeostasis [21]. Some pharmacological studies have already been performed [110] or are ongoing [21], and preliminary results are interesting and may lead to further investigation.

AD is the most common neurodegenerative disorder in the Western world, affecting 1 person out of 9 aged 65 and older [111] but still lacks an effective treatment. The investigation of LC involvement through LC-MRI may significantly enhance our understanding of its pathogenesis, facilitating precise diagnostic approaches and the development of novel disease-modifying therapies.

## References

[1] Ehrenberg AJ, Kelberman MA, Liu KY, Dahl MJ, Weinshenker D, Falgàs N, Dutt S, Mather M, Ludwig M, Betts MJ (2023). Priorities for research on neuromodulatory subcortical systems in Alzheimer’s disease: position paper from the NSS PIA of ISTAART. Alzheimers Dement.

[2] Poe GR, Foote S, Eschenko O, Johansen JP, Bouret S, Aston-Jones G, Harley CW, Manahan-Vaughan D, Weinshenker D, Valentino R (2020). Locus coeruleus: a new look at the blue spot. Nat Rev Neurosci.

[3] Sara SJ (2009). The locus coeruleus and noradrenergic modulation of cognition. Nat Rev Neurosci.

[4] Moruzzi G, Magoun HW (1995). Brain stem reticular formation and activation of the EEG. 1949. J Neuropsychiatry Clin Neurosci..

[5] Aston-Jones G, Cohen JD (2005). An integrative theory of locus coeruleus-norepinephrine function: adaptive gain and optimal performance. Annu Rev Neurosci.

[6] Berridge CW, Waterhouse BD (2003). The locus coeruleus-noradrenergic system: modulation of behavioral state and state-dependent cognitive processes. Brain Res Brain Res Rev.

[7] Giorgi FS, Galgani A, Puglisi-Allegra S, Limanaqi F, Busceti CL, Fornai F (2020). Locus coeruleus and neurovascular unit: from its role in physiology to its potential role in Alzheimer’s disease pathogenesis. J Neurosci Res.

[8] Giorgi FS, Saccaro LF, Galgani A, Busceti CL, Biagioni F, Frati A, Fornai F (2019). The role of locus coeruleus in neuroinflammation occurring in Alzheimer’s disease. Brain Res Bull.

[9] Fernandes P, Regala J, Correia F, Gonçalves-Ferreira AJ (2012). The human locus coeruleus 3-D stereotactic anatomy. Surg Radiol Anat.

[10] Weinshenker D (2018). Long road to ruin: noradrenergic dysfunction in neurodegenerative disease. Trends Neurosci.

[11] Kelly SC, Nelson PT, Counts SE (2021). Pontine arteriolosclerosis and locus coeruleus oxidative stress differentiate resilience from mild cognitive impairment in a clinical pathologic cohort. J Neuropathol Exp Neurol.

[12] Manaye KF, McIntire DD, Mann DM, German DC (1995). Locus coeruleus cell loss in the aging human brain: a non-random process. J Comp Neurol.

[13] Braak H, Thal DR, Ghebremedhin E, Del Tredici K (2011). Stages of the pathologic process in Alzheimer disease: age categories from 1 to 100 years. J Neuropathol Exp Neurol.

[14] Theofilas P, Ehrenberg AJ, Dunlop S, Di Lorenzo Alho AT, Nguy A, Leite REP, Rodriguez RD, Mejia MB, Suemoto CK, Ferretti-Rebustini REL (2017). Locus coeruleus volume and cell population changes during Alzheimer’s disease progression: a stereological study in human postmortem brains with potential implication for early-stage biomarker discovery. Alzheimers Dement.

[15] Giorgi FS, Ryskalin L, Ruffoli R, Biagioni F, Limanaqi F, Ferrucci M, Busceti CL, Bonuccelli U, Fornai F (2017). The neuroanatomy of the reticular nucleus locus coeruleus in Alzheimer’s disease. Front Neuroanat.

[16] Heneka MT, Ramanathan M, Jacobs AH, Dumitrescu-Ozimek L, Bilkei-Gorzo A, Debeir T, Sastre M, Galldiks N, Zimmer A, Hoehn M (2006). Locus ceruleus degeneration promotes Alzheimer pathogenesis in amyloid precursor protein 23 transgenic mice. J Neurosci.

[17] Heneka MT, Nadrigny F, Regen T, Martinez-Hernandez A, Dumitrescu-Ozimek L, Terwel D, Jardanhazi-Kurutz D, Walter J, Kirchhoff F, Hanisch UK, Kummer MP (2010). Locus ceruleus controls Alzheimer’s disease pathology by modulating microglial functions through norepinephrine. Proc Natl Acad Sci U S A.

[18] Jardanhazi-Kurutz D, Kummer MP, Terwel D, Vogel K, Dyrks T, Thiele A, Heneka MT (2010). Induced LC degeneration in APP/PS1 transgenic mice accelerates early cerebral amyloidosis and cognitive deficits. Neurochem Int.

[19] Kalinin S, Feinstein DL, Xu HL, Huesa G, Pelligrino DA, Galea E (2006). Degeneration of noradrenergic fibres from the locus coeruleus causes tight-junction disorganisation in the rat brain. Eur J Neurosci.

[20] Beardmore R, Hou R, Darekar A, Holmes C, Boche D (2021). The locus coeruleus in aging and Alzheimer’s disease: a postmortem and brain imaging review. J Alzheimers Dis.

[21] David M, Malhotra PA (2022). New approaches for the quantification and targeting of noradrenergic dysfunction in Alzheimer’s disease. Ann Clin Transl Neurol.

[22] Betts MJ, Kirilina E, Otaduy MCG, Ivanov D, Acosta-Cabronero J, Callaghan MF, Lambert C, Cardenas-Blanco A, Pine K, Passamonti L (2019). Locus coeruleus imaging as a biomarker for noradrenergic dysfunction in neurodegenerative diseases. Brain.

[23] Keren NI, Lozar CT, Harris KC, Morgan PS, Eckert MA (2009). In vivo mapping of the human locus coeruleus. Neuroimage.

[24] Shibata E, Sasaki M, Tohyama K, Kanbara Y, Otsuka K, Ehara S, Sakai A (2006). Age-related changes in locus ceruleus on neuromelanin magnetic resonance imaging at 3 Tesla. Magn Reson Med Sci.

[25] Zucca FA, Bellei C, Giannelli S, Terreni MR, Gallorini M, Rizzio E, Pezzoli G, Albertini A, Zecca L (2006). Neuromelanin and iron in human locus coeruleus and substantia nigra during aging: consequences for neuronal vulnerability. J Neural Transm (Vienna).

[26] Chen X, Huddleston DE, Langley J, Ahn S, Barnum CJ, Factor SA, Levey AI, Hu X (2014). Simultaneous imaging of locus coeruleus and substantia nigra with a quantitative neuromelanin MRI approach. Magn Reson Imaging.

[27] Dixon WT, Engels H, Castillo M, Sardashti M (1990). Incidental magnetization transfer contrast in standard multislice imaging. Magn Reson Imaging.

[28] Sled JG (2018). Modelling and interpretation of magnetization transfer imaging in the brain. Neuroimage.

[29] Keren NI, Taheri S, Vazey EM, Morgan PS, Granholm AC, Aston-Jones GS, Eckert MA (2015). Histologic validation of locus coeruleus MRI contrast in post-mortem tissue. Neuroimage.

[30] Trujillo P, Petersen KJ, Cronin MJ, Lin YC, Kang H, Donahue MJ, Smith SA, Claassen DO (2019). Quantitative magnetization transfer imaging of the human locus coeruleus. Neuroimage.

[31] Watanabe T, Tan Z, Wang X, Martinez-Hernandez A, Frahm J (2019). Magnetic resonance imaging of noradrenergic neurons. Brain Struct Funct.

[32] Clewett DV, Lee TH, Greening S, Ponzio A, Margalit E, Mather M (2016). Neuromelanin marks the spot: identifying a locus coeruleus biomarker of cognitive reserve in healthy aging. Neurobiol Aging.

[33] Hämmerer D, Callaghan MF, Hopkins A, Kosciessa J, Betts M, Cardenas-Blanco A, Kanowski M, Weiskopf N, Dayan P, Dolan RJ, Düzel E (2018). Locus coeruleus integrity in old age is selectively related to memories linked with salient negative events. Proc Natl Acad Sci U S A.

[34] Betts MJ, Cardenas-Blanco A, Kanowski M, Jessen F, Düzel E (2017). In vivo MRI assessment of the human locus coeruleus along its rostrocaudal extent in young and older adults. Neuroimage.

[35] Galgani A, Bartolini E, D'Amora M, Faraguna U, Giorgi FS (2023). The central noradrenergic system in neurodevelopmental disorders: merging experimental and clinical evidence. Int J Mol Sci.

[36] Galgani A, Lombardo F, Della Latta D, Martini N, Bonuccelli U, Fornai F, Giorgi FS (2020). Locus coeruleus magnetic resonance imaging in neurological diseases. Curr Neurol Neurosci Rep.

[37] Liu KY, Marijatta F, Hämmerer D, Acosta-Cabronero J, Düzel E, Howard RJ (2017). Magnetic resonance imaging of the human locus coeruleus: a systematic review. Neurosci Biobehav Rev.

[38] Giorgi FS, Martini N, Lombardo F, Galgani A, Bastiani L, Della Latta D, Hlavata H, Busceti CL, Biagioni F, Puglisi-Allegra S (2022). Locus Coeruleus magnetic resonance imaging: a comparison between native-space and template-space approach. J Neural Transm (Vienna).

[39] Dahl MJ, Mather M, Düzel S, Bodammer NC, Lindenberger U, Kühn S, Werkle-Bergner M (2019). Rostral locus coeruleus integrity is associated with better memory performance in older adults. Nat Hum Behav.

[40] Liu KY, Acosta-Cabronero J, Cardenas-Blanco A, Loane C, Berry AJ, Betts MJ, Kievit RA, Henson RN, Düzel E, Howard R, Hämmerer D, Cam-CAN (2019). In vivo visualization of age-related differences in the locus coeruleus. Neurobiol Aging..

[41] Liu KY, Kievit RA, Tsvetanov KA, Betts MJ, Düzel E, Rowe JB, Howard R, Hämmerer D, Cam-CAN (2020). Noradrenergic-dependent functions are associated with age-related locus coeruleus signal intensity differences. Nat Commun..

[42] Giorgi FS, Lombardo F, Galgani A, Hlavata H, Della Latta D, Martini N, Pavese N, Ghicopulos I, Baldacci F, Coi A (2022). Locus coeruleus magnetic resonance imaging in cognitively intact elderly subjects. Brain Imaging Behav.

[43] Bachman SL, Dahl MJ, Werkle-Bergner M, Düzel S, Forlim CG, Lindenberger U, Kühn S, Mather M (2021). Locus coeruleus MRI contrast is associated with cortical thickness in older adults. Neurobiol Aging.

[44] Van Egroo M, van Hooren RWE, Jacobs HIL (2021). Associations between locus coeruleus integrity and nocturnal awakenings in the context of Alzheimer’s disease plasma biomarkers: a 7T MRI study. Alzheimers Res Ther.

[45] Elman JA, Puckett OK, Hagler DJ, Pearce RC, Fennema-Notestine C, Hatton SN, Lyons MJ, McEvoy LK, Panizzon MS, Reas ET (2022). Associations between MRI-assessed locus coeruleus integrity and cortical gray matter microstructure. Cereb Cortex.

[46] Bell TR, Elman JA, Beck A, Fennema-Notestine C, Gustavson DE, Hagler DJ, Jack AJ, Lyons MJ, Puckett OK, Toomey R (2023). Rostral-middle locus coeruleus integrity and subjective cognitive decline in early old age. J Int Neuropsychol Soc.

[47] Al Haddad R, Chamoun M, Tardif CL, Guimond S, Horga G, Rosa-Neto P, Cassidy CM (2023). Normative values of neuromelanin-sensitive MRI signal in older adults obtained using a turbo spin echo sequence. J Magn Reson Imaging.

[48] Van Egroo M, Riphagen JM, Ashton NJ, Janelidze S, Sperling RA, Johnson KA, Yang HS, Bennett DA, Blennow K, Hansson O (2023). Ultra-high field imaging, plasma markers and autopsy data uncover a specific rostral locus coeruleus vulnerability to hyperphosphorylated tau. Mol Psychiatry.

[49] Dahl MJ, Bachman SL, Dutt S, Düzel S, Bodammer NC, Lindenberger U, Kühn S, Werkle-Bergner M, Mather M (2023). The integrity of dopaminergic and noradrenergic brain regions is associated with different aspects of late-life memory performance. Nat Aging.

[50] Langley J, Hussain S, Huddleston DE, Bennett IJ, Hu XP (2022). Impact of locus coeruleus and its projections on memory and aging. Brain Connect.

[51] Porat S, Sibilia F, Yoon J, Shi Y, Dahl MJ, Werkle-Bergner M, Düzel S, Bodammer N, Lindenberger U, Kühn S, Mather M (2022). Age differences in diffusivity in the locus coeruleus and its ascending noradrenergic tract. Neuroimage.

[52] Plini ERG, O'Hanlon E, Boyle R, Sibilia F, Rikhye G, Kenney J, Whelan R, Melnychuk MC, Robertson IH, Dockree PM (2021). Examining the role of the noradrenergic locus coeruleus for predicting attention and brain maintenance in healthy old age and disease: an MRI structural study for the Alzheimer’s disease neuroimaging initiative. Cells.

[53] Berger A, Koshmanova E, Beckers E, Sharifpour R, Paparella I, Campbell I, Mortazavi N, Balda F, Yi YJ, Lamalle L (2023). Structural and functional characterization of the locus coeruleus in young and late middle-aged individuals. Front Neuroimaging.

[54] Veréb D, Mijalkov M, Canal-Garcia A, Chang YW, Gomez-Ruiz E, Gerboles BZ, Kivipelto M, Svenningsson P, Zetterberg H, Volpe G (2023). Age-related differences in the functional topography of the locus coeruleus and their implications for cognitive and affective functions. Elife..

[55] •• Jacobs HIL, Becker JA, Kwong K, Engels-Domínguez N, Prokopiou PC, Papp KV, Properzi M, Hampton OL, d'Oleire Uquillas F, Sanchez JS et al. In vivo and neuropathology data support locus coeruleus integrity as indicator of Alzheimer’s disease pathology and cognitive decline. Sci Transl Med. 2021; 13(612):eabj2511. 10.1126/scitranslmed.abj2511. **In this report, for the first time, the locus coeruleus MRI-signal was put in relation with in vivo amyloid and tau biomarkers in a well-selected cohort of cognitively unimpaired and impaired older individuals with longitudinal cognitive measures.** 10.1126/scitranslmed.abj2511 PMC8641759 34550726

[56] Miyoshi F, Ogawa T, Kitao SI, Kitayama M, Shinohara Y, Takasugi M, Fujii S, Kaminou T (2013). Evaluation of Parkinson disease and Alzheimer disease with the use of neuromelanin MR imaging and (123)I-metaiodobenzylguanidine scintigraphy. AJNR Am J Neuroradiol.

[57] Takahashi J, Shibata T, Sasaki M, Kudo M, Yanezawa H, Obara S, Kudo K, Ito K, Yamashita F, Terayama Y (2015). Detection of changes in the locus coeruleus in patients with mild cognitive impairment and Alzheimer’s disease: high-resolution fast spin-echo T1-weighted imaging. Geriatr Gerontol Int.

[58] Dordevic M, Müller-Fotti A, Müller P, Schmicker M, Kaufmann J, Müller NG (2017). Optimal cut-off value for locus coeruleus-to-pons intensity ratio as clinical biomarker for Alzheimer’s disease: a pilot study. J Alzheimers Dis Rep.

[59] Betts MJ, Cardenas-Blanco A, Kanowski M, Spottke A, Teipel SJ, Kilimann I, Jessen F, Düzel E (2019). Locus coeruleus MRI contrast is reduced in Alzheimer’s disease dementia and correlates with CSF Aβ levels. Alzheimers Dement (Amst).

[60] Olivieri P, Lagarde J, Lehericy S, Valabrègue R, Michel A, Macé P, Caillé F, Gervais P, Bottlaender M, Sarazin M (2019). Early alteration of the locus coeruleus in phenotypic variants of Alzheimer’s disease. Ann Clin Transl Neurol.

[61] Hou R, Beardmore R, Holmes C, Osmond C, Darekar A (2021). A case-control study of the locus coeruleus degeneration in Alzheimer’s disease. Eur Neuropsychopharmacol.

[62] Dutt S, Li Y, Mather M, Nation DA, Alzheimer’s disease neuroimaging initiative (2020). Brainstem volumetric integrity in preclinical and prodromal Alzheimer’s disease. J Alzheimers Dis..

[63] Cassidy CM, Therriault J, Pascoal TA, Cheung V, Savard M, Tuominen L, Chamoun M, McCall A, Celebi S, Lussier F (2022). Association of locus coeruleus integrity with Braak stage and neuropsychiatric symptom severity in Alzheimer’s disease. Neuropsychopharmacology.

[64] Jacobs HIL, Becker JA, Kwong K, Munera D, Ramirez-Gomez L, Engels-Domínguez N, Sanchez JS, Vila-Castelar C, Baena A, Sperling RA (2023). Waning locus coeruleus integrity precedes cortical tau accrual in preclinical autosomal dominant Alzheimer’s disease. Alzheimers Dement.

[65] Li M, Liu S, Zhu H, Guo Z, Zhi Y, Liu R, Jiang Z, Liang X, Hu H, Zhu J (2022). Decreased locus coeruleus signal associated with Alzheimer’s disease based on neuromelanin-sensitive magnetic resonance imaging technique. Front Neurosci.

[66] Dahl MJ, Mather M, Werkle-Bergner M, Kennedy BL, Guzman S, Hurth K, Miller CA, Qiao Y, Shi Y, Chui HC, Ringman JM (2022). Locus coeruleus integrity is related to tau burden and memory loss in autosomal-dominant Alzheimer’s disease. Neurobiol Aging.

[67] Aghakhanyan G, Galgani A, Vergallo A, Lombardo F, Martini N, Baldacci F, Tognoni G, Leo A, Guidoccio F, Siciliano G (2023). Brain metabolic correlates of locus coeruleus degeneration in Alzheimer’s disease: a multimodal neuroimaging study. Neurobiol Aging.

[68] Liu R, Guo Z, Li M, Liu S, Zhi Y, Jiang Z, Liang X, Hu H, Zhu J. Lower fractional dimension in Alzheimer’s disease correlates with reduced locus coeruleus signal intensity. Magn Reson Imaging. 2023;2:S0730–725X(23)00146–7. 10.1016/j.mri.2023.08.001. 10.1016/j.mri.2023.08.001 37541457

[69] Galgani A, Lombardo F, Martini N, Vergallo A, Bastiani L, Hampel H, Hlavata H, Baldacci F, Tognoni G, De Marchi D (2023). Magnetic resonance imaging locus coeruleus abnormality in amnestic mild cognitive impairment is associated with future progression to dementia. Eur J Neurol.

[70] Galgani A, Palermo G, Lombardo F, Martini N, Bastiani L, Vergallo A, Tommasini L, Bellini G, Baldacci F, Frosini D (2023). Different patterns of locus coeruleus MRI alteration in Alzheimer’s and dementia with Lewy bodies. Curr Alzheimer Res.

[71] Elman JA, Puckett OK, Beck A, Fennema-Notestine C, Cross LK, Dale AM, Eglit GML, Eyler LT, Gillespie NA, Granholm EL (2021). MRI-assessed locus coeruleus integrity is heritable and associated with multiple cognitive domains, mild cognitive impairment, and daytime dysfunction. Alzheimers Dement.

[72] Kelly SC, He B, Perez SE, Ginsberg SD, Mufson EJ, Counts SE (2017). Locus coeruleus cellular and molecular pathology during the progression of Alzheimer’s disease. Acta Neuropathol Commun.

[73] Szabadi E. Functional neuroanatomy of the central noradrenergic system. J Psychopharmacol. 2013; 27(8):659-93. 10.1177/0269881113490326. Erratum in: J Psychopharmacol. 2013; 27(10):964. 10.1177/0269881113490326 23761387

[74] Schwarz LA, Luo L (2015). Organization of the locus coeruleus-norepinephrine system. Curr Biol.

[75] Braak H, Braak E (1991). Neuropathological stageing of Alzheimer-related changes. Acta Neuropathol.

[76] Braak H, Braak E. Staging of Alzheimer’s disease-related neurofibrillary changes. Neurobiol Aging. 1995; 16(3):271–8; discussion 278–84. 10.1016/0197-4580(95)00021-6. 10.1016/0197-4580(95)00021-6 7566337

[77] Igarashi KM (2023). Entorhinal cortex dysfunction in Alzheimer’s disease. Trends Neurosci.

[78] Tang Y, Cao M, Li Y, Lin Y, Wu X, Chen M, Alzheimer’s disease neuroimaging initiative (2023). Altered structural covariance of locus coeruleus in individuals with significant memory concern and patients with mild cognitive impairment. Cereb Cortex..

[79] Dubois B, Feldman HH, Jacova C, Hampel H, Molinuevo JL, Blennow K, DeKosky ST, Gauthier S, Selkoe D, Bateman R, et al. Advancing research diagnostic criteria for Alzheimer’s disease: the IWG-2 criteria. Lancet Neurol. 2014; 13(6):614-29. 10.1016/S1474-4422(14)70090-0. Erratum in: Lancet Neurol. 2014; 13(8):757. 10.1016/S1474-4422(14)70090-0 24849862

[80] Prokopiou PC, Engels-Domínguez N, Schultz AP, Sepulcre J, Koops EA, Papp KV, Marshall GA, Normandin MD, El Fakhri G, Rentz D (2023). Association of novelty-related locus coeruleus function with entorhinal tau deposition and memory decline in preclinical Alzheimer disease. Neurology.

[81] Prokopiou PC, Engels-Domínguez N, Papp KV, Scott MR, Schultz AP, Schneider C, Farrell ME, Buckley RF, Quiroz YT, El Fakhri G (2022). Lower novelty-related locus coeruleus function is associated with Aβ-related cognitive decline in clinically healthy individuals. Nat Commun.

[82] Braak H, Del Tredici K (2011). Alzheimer’s pathogenesis: is there neuron-to-neuron propagation?. Acta Neuropathol.

[83] Kang SS, Liu X, Ahn EH, Xiang J, Manfredsson FP, Yang X, Luo HR, Liles LC, Weinshenker D, Ye K (2020). Norepinephrine metabolite DOPEGAL activates AEP and pathological tau aggregation in locus coeruleus. J Clin Invest.

[84] Kang SS, Meng L, Zhang X, Wu Z, Mancieri A, Xie B, Liu X, Weinshenker D, Peng J, Zhang Z, Ye K (2022). Tau modification by the norepinephrine metabolite DOPEGAL stimulates its pathology and propagation. Nat Struct Mol Biol.

[85] Arnsten AFT, Datta D, Del Tredici K, Braak H (2021). Hypothesis: tau pathology is an initiating factor in sporadic Alzheimer’s disease. Alzheimers Dement.

[86] Iba M, McBride JD, Guo JL, Zhang B, Trojanowski JQ, Lee VM (2015). Tau pathology spread in PS19 tau transgenic mice following locus coeruleus (LC) injections of synthetic tau fibrils is determined by the LC’s afferent and efferent connections. Acta Neuropathol.

[87] Chalermpalanupap T, Schroeder JP, Rorabaugh JM, Liles LC, Lah JJ, Levey AI, Weinshenker D (2018). Locus coeruleus ablation exacerbates cognitive deficits, neuropathology, and lethality in P301S tau transgenic mice. J Neurosci.

[88] Ehrenberg AJ, Nguy AK, Theofilas P, Dunlop S, Suemoto CK, Di Lorenzo Alho AT, Leite RP, Diehl Rodriguez R, Mejia MB, Rüb U (2017). Quantifying the accretion of hyperphosphorylated tau in the locus coeruleus and dorsal raphe nucleus: the pathological building blocks of early Alzheimer’s disease. Neuropathol Appl Neurobiol.

[89] Giorgi FS, Biagioni F, Galgani A, Pavese N, Lazzeri G, Fornai F (2020). Locus coeruleus modulates neuroinflammation in parkinsonism and dementia. Int J Mol Sci.

[90] Montagne A, Zhao Z, Zlokovic BV (2017). Alzheimer’s disease: a matter of blood-brain barrier dysfunction?. J Exp Med.

[91] Scheltens P, Blennow K, Breteler MM, de Strooper B, Frisoni GB, Salloway S, Van der Flier WM (2016). Alzheimer’s disease. Lancet.

[92] Bast N, Poustka L, Freitag CM (2018). The locus coeruleus-norepinephrine system as pacemaker of attention - a developmental mechanism of derailed attentional function in autism spectrum disorder. Eur J Neurosci.

[93] Aston-Jones G, Rajkowski J, Cohen J (1999). Role of locus coeruleus in attention and behavioral flexibility. Biol Psychiatry.

[94] Malhotra PA (2019). Impairments of attention in Alzheimer’s disease. Curr Opin Psychol.

[95] Hansen N (2017). The longevity of hippocampus-dependent memory is orchestrated by the locus coeruleus-noradrenergic system. Neural Plast.

[96] Coradazzi M, Gulino R, Fieramosca F, Falzacappa LV, Riggi M, Leanza G (2016). Selective noradrenaline depletion impairs working memory and hippocampal neurogenesis. Neurobiol Aging.

[97] Hou L, Sun F, Sun W, Zhang L, Wang Q (2019). Lesion of the locus coeruleus damages learning and memory performance in Paraquat and Maneb-induced mouse Parkinson’s disease model. Neuroscience.

[98] Bacon TJ, Pickering AE, Mellor JR (2020). Noradrenaline release from locus coeruleus terminals in the hippocampus enhances excitation-spike coupling in CA1 pyramidal neurons via β-adrenoceptors. Cereb Cortex.

[99] Hansen N, Manahan-Vaughan D (2015). Locus coeruleus stimulation facilitates long-term depression in the dentate gyrus that requires activation of β-adrenergic receptors. Cereb Cortex.

[100] Zhao QF, Tan L, Wang HF, Jiang T, Tan MS, Tan L, Xu W, Li JQ, Wang J, Lai TJ, Yu JT (2016). The prevalence of neuropsychiatric symptoms in Alzheimer’s disease: systematic review and meta-analysis. J Affect Disord.

[101] McCall JG, Siuda ER, Bhatti DL, Lawson LA, McElligott ZA, Stuber GD, Bruchas MR (2017). Locus coeruleus to basolateral amygdala noradrenergic projections promote anxiety-like behavior. Elife.

[102] Borodovitsyna O, Flamini MD, Chandler DJ (2018). Acute stress persistently alters locus coeruleus function and anxiety-like behavior in adolescent rats. Neuroscience.

[103] Weiss JM, Stout JC, Aaron MF, Quan N, Owens MJ, Butler PD, Nemeroff CB (1994). Depression and anxiety: role of the locus coeruleus and corticotropin-releasing factor. Brain Res Bull.

[104] Guinea-Izquierdo A, Giménez M, Martínez-Zalacaín I, Del Cerro I, Canal-Noguer P, Blasco G, Gascón J, Reñé R, Rico I, Camins A (2021). Lower locus coeruleus MRI intensity in patients with late-life major depression. PeerJ.

[105] Calarco N, Cassidy CM, Selby B, Hawco C, Voineskos AN, Diniz BS, Nikolova YS (2022). Associations between locus coeruleus integrity and diagnosis, age, and cognitive performance in older adults with and without late-life depression: an exploratory study. Neuroimage Clin.

[106] Van Egroo M, Koshmanova E, Vandewalle G, Jacobs HIL (2022). Importance of the locus coeruleus-norepinephrine system in sleep-wake regulation: implications for aging and Alzheimer’s disease. Sleep Med Rev.

[107] Koshmanova E, Berger A, Beckers E, Campbell I, Mortazavi N, Sharifpour R, Paparella I, Balda F, Berthomier C, Degueldre C, et al. Locus coeruleus activity while awake is associated with REM sleep quality in older individuals. JCI Insight. 2023;e172008. 10.1172/jci.insight.172008. 10.1172/jci.insight.172008 PMC10619502 37698926

[108] Musiek ES, Xiong DD, Holtzman DM (2015). Sleep, circadian rhythms, and the pathogenesis of Alzheimer disease. Exp Mol Med.

[109] Giorgi FS, Galgani A, Puglisi-Allegra S, Busceti CL, Fornai F (2021). The connections of locus coeruleus with hypothalamus: potential involvement in Alzheimer’s disease. J Neural Transm (Vienna).

[110] Levey AI, Qiu D, Zhao L, Hu WT, Duong DM, Higginbotham L, Dammer EB, Seyfried NT, Wingo TS, Hales CM (2022). A phase II study repurposing atomoxetine for neuroprotection in mild cognitive impairment. Brain.

[111] 2023 Alzheimer’s disease facts and figures. Alzheimers Dement. 2023; 19(4):1598-1695. 10.1002/alz.13016 10.1002/alz.13016 36918389

